# Therapeutic effects of astragaloside IV and *Astragalus spinosus saponins* against bisphenol A-induced neurotoxicity and DNA damage in rats

**DOI:** 10.7717/peerj.11930

**Published:** 2021-08-09

**Authors:** Amina E. Essawy, Heba-Tallah Abd Elrahim Abd Elkader, Omaima A. Khamiss, Saber Mohamed Eweda, Heba Mohamed Abdou

**Affiliations:** 1 Zoology Department, Faculty of Science, Alexandria University, Alexandria, Egypt; 2 Zoology, Biological and Geological Sciences Department, Faculty of Education, Alexandria University, Alexandria, Egypt; 3 Animal Biotechnology Department, Genetic Engineering and Biotechnology Research Institute (GEBRI-USC), University of Sadat City, Sadat City, Egypt; 4 Department of Biochemistry, Faculty of Science, Alexandria University, Alexandria, Egypt; 5 Department of Medical Laboratories Technology, College of Applied Medical Sciences, Taibah University, Madinah, KSA, Saudi Arabia

**Keywords:** ASIV, * A. spinosus*, BPA, Neurotoxicity, DNA damage

## Abstract

**Background:**

Bisphenol A (BPA) is an endocrine disruptor to which humans are often subjected during daily life. This study aimed to investigate the ameliorative effect of astragaloside IV (ASIV) or saponins extracted from *Astragalus spinosus* (*A. spinosus*) against DNA damage and neurotoxic effects induced by BPA in prefrontal cortex (PFC), hippocampal and striatal brain regions of developing male rats.

**Materials and Methods:**

Juvenile PND20 (pre-weaning; age of 20 days) male Sprague Dawley rats were randomly and equally divided into four groups: control, BPA, BPA+ASIV and BPA+*A. spinosus* saponins groups. Bisphenol A (125 mg/kg/day) was administrated orally to male rats from day 20 (BPA group) and along with ASIV (80 mg/kg/day) (BPA+ASIV group) or *A. spinosus* saponin (100 mg/kg/day) (BPA+ *A. spinosus* saponins group) from day 50 to adult age day 117.

**Results:**

Increased level of nitric oxide (NO) and decreased level of glutamate (Glu), glutamine (Gln), glutaminase (GA) and glutamine synthetase (GS) were observed in the brain regions of BPA treated rats compared with the control. On the other hand, co-administration of ASIV or *A. spinosus* saponin with BPA considerably improved levels of these neurochemicals. The current study also revealed restoration of the level of brain derived neurotrophic factor (BDNF) and N-methyl-D-aspartate receptors (NR_2_A and NR_2_B) gene expression in BPA+ ASIV and BPA+*A. spinosus* saponins groups. The co-treatment of BPA group with ASIV or *A. spinosus* saponin significantly reduced the values of comet parameters as well as the intensity of estrogen receptors (ERs) immunoreactive cells and improved the histological alterations induced by BPA in different brain regions.

**Conclusion:**

It could be concluded that ASIV or *A. spinosus* saponins has a promising role in modulating the neurotoxicity and DNA damage elicited by BPA.

## Introduction

The intervention in the early developmental stages of the central nervous system (CNS) produces permanent alterations in the brain physiology in different life stages of the exposed animals and their progeny (Manikkam et al., 2013). Early BPA exposure causes a broad spectrum of neurodevelopmental diseases, which might be due to disturbance in neurogenesis, synaptogenesis, and myelination (Iser et al., 2016). Several studies linked the neurotoxic effects of BPA to its potency to perturb the signaling of estrogen receptor (ER) in developing neurons. This perturbation leading to massive proliferation and differentiation of neurons, producing endogenous estrogen, disruption in nuclear translocation of estrogen receptors (ER) and modifying the genetic and epigenetic programming (Chung et al., 2011; Delfosse et al., 2012; Kundakovic et al., 2013; Xu et al., 2014).

Astragaloside IV (ASIV), the main bioactive constituent in *Astragalus* sp., was distinguished by the presence of 3, 6 and/or 25-coupled glucose moieties and has a molecular formula C_14_H_68_O_14_ (Ionkova et al., 2014). ASIV exhibited various biological activities including anti-inflammatory antioxidant, antilipolytic and antiapoptotic (Lu et al., 2015). It has been reported that ASIV reduced the production of nitric oxide (NO) in brain tissues and enhanced the permeability of blood brain barrier (BBB) following ischemia (Huang et al., 2017). Wu et al. (2018) showed that ASIV, the main constituent of *Astragalus membranaceus* leaf extract, has anxiolytic, anti-depression, and anti-inflammatory effect in immobilization stress-induced anxiety in mice.

*Astragalus spinosus* (*A. spinosus*) is a flowering plant from the largest familiar genera in the Fabaceae family. Saponins, widely investigated secondary metabolites, are fundamental biologically active constituents of *A. spinosus* (Yang et al., 2013). Saponins attenuate the proliferation of neural progenitor stem cells, induce the expression of nerve growth-related factors, facilitate neuron regeneration and improve memory and learning (Hu et al., 2014). Moreover, *Astragalus* saponins exhibited neuroprotective potential that could be attributed to their antioxidant, anti-apoptotic and anti-depressive effects (Kamal, Arif & Jawaid, 2017). The present study was designed to investigate the role of ASIV and saponins extracted from *A. spinosus* against BPA-induced neurotoxicity and DNA damage in the brain of the developing male rat.

## Materials and Methods

### Experimental animals and study design

A total of 64 juvenile (PND20) male Sprague Dawley rats weighing 25–38 gm were reared in the Faculty of Agriculture, Alexandria University, Alexandria, Egypt and used in the current study. The rats were housed in stainless steel cages with free access to standard pellet diet and tap water *ad libitum*. Rats were maintained under 50–60% relative humidity and 25 ± 5 °C with a 12 h light/dark cycle. After acclimatization, rats were randomly and equally divided into four groups, 16 rats in each. Rats of the first group served as control and were administered orally with 0.5 ml corn oil. Each rat of the second group was force-fed with BPA (Loba Chemie for Laboratory Reagents and Fine Chemicals, India) at a dose of 125 mg/kg/day dissolved in corn oil (Ahbab, Barlas & Karabulut, 2017). Rats in the third (BPA+ASIV) and fourth (BPA+ *A. spinosus* saponins) groups received BPA (125 mg/kg/day) from PND20 and co-treated with ASIV (98%) (Eyechem, Xi’an QingShuo Import and Export Trade, China) at a dose of 80 mg/kg/day (Lu et al., 2015) or *A. spinosus* saponin at a dose of 100mg/kg/day (Bahaeddin et al., 2016) respectively from PND50 to PND117. Saponin was extracted from the shoot system of *Astragalus spinosus* collected from Al-Ameed Nature Reserve, Alexandria, Egypt. The extraction was carried out according to the method described by Shawky & Selim (2017) and Abd Elkader et al. (2021). The experimental protocol and techniques of the current study were reviewed and approved by Institutional Animal Care and Use Committee of Alexandria University (ALEXU-IACUC), a member of the International Council for Laboratory Animal Science (ICLAS) (Approval number: AU04200125201). The methods were carried out in accordance with the approved guidelines and all efforts were made to minimize the suffering of rats during the experimental period.

### Sampling and preparation of brain tissue

At the end of experimental period (PND117), rats were fasted overnight. The overnight fasting period prior to sampling did not cause remarkable loss of body weight or any observed adverse effects on rats. Rats were sacrificed under light anesthesia followed by cervical dislocation. All efforts were made to minimize the suffering of rats during the experimental period. The brain tissues of 52 rats, 13 from each group, were quickly removed, washed with cold normal saline, and dissected to isolate the PFC, hippocampus, and striatum regions. The distinct brain regions of seven rats from each group were used for the assessment of biochemical parameters. The brain regions of the other six animals of each group were partitioned into two equal halves for molecular and comet assays. All the brain samples were stored at −80 °C until use. For histopathological and immunohistochemical examination, three rats from each group were sacrificed followed by intracardiac perfusion with cold saline solution and brain samples from each group were promptly fixed in 10% formalin.

### Determination of biochemical parameters in the isolated brain regions

Brain regions were homogenized in 20 times (w/v) ice-cold TES buffer (25 mM Tris–HCl, 0.2 mM EDTA and 0.33 M sucrose, pH = 7.4) with a protease inhibitor cocktail (SRE0055, Sigma-Aldrich, Germany). The homogenates were centrifuged at 1800 xg for 10 min at 4 °C (Hettich zentrifugen, Universal 32 R; Hettich Lab. Tuttlingen, Germany). Total protein content was measured according to the method of Lowry et al. (1951). Nitric oxide (NO) concentration in the supernatant of different brain regions was estimated colorimetrically as nitrite according to the method of Montgomery & Dymock (1961) using a standard commercial kit from Biodiagnostic Company (Cairo, Egypt). The specific activity of glutaminase (GA) in the brain regions was assayed according to the procedure described in Blanco et al. (2015). Glutamine synthetase (GS) activity was assayed in brain regions according to the modified method of Momosaki et al. (2015) and expressed as mM of γ-glutamyl hydroxamate/min/mg protein. The concentration of glutamate (Glu) in different brain regions was measured enzymatically using glutamate dehydrogenase according to the method of Bernt & Bergmeyer (1974). Glutamine (Gln) levels were determined using purified glutaminase from *E. coli* (Sigma-Aldrich, Germany) according to the methods described by Lund & Bergmeyer (1985). The brain derived neurotrophic factor **(**BDNF) concentration was estimated using BDNF microplate assay kit (Shanghai Sunred Biological Technology, South Korea) according to the manufacturer’s protocol.

### Molecular analysis and gene expression of NMDA ionotropic receptors in brain regions

Quantitative real-time RT-PCR was carried out for relative quantification of the mRNA gene expression level of NMDA ionotropic receptors subunits (NR_2_A and NR_2_B) in the PFC, hippocampal, and striatal brain regions. Total RNA was extracted using TRIzol reagent (Invitrogen, cat no. 15596-026). Details on the RNA extraction and cDNA synthesis have been previously described (Blanco et al., 2015). The synthesized cDNAs were amplified separately through 50 cycles with specific primer for NR_2_A, NR_2_B and β-actin using SYBR Green with Low ROX according to the manufacturer’s recommended conditions. The primer sequences and melting temperatures used are described in Table 1. The housekeeping gene, β-actin, was used as a reference standard to normalize the data. The relative expression of genes was determined using the 2^−ΔΔCT^ method with normalization to β-actin expression.

**Table 1:** The primer sequences, accession number and melting temperatures of β-actin, NR_2_A and NR_2_B.

**Target gene**	**Primers with sequences**	**Accession number**		**Tm**
**β-actin**	**Sense:** 5′-GGAGATTACTGCCCTGGCTCCTA-3 **Anti-sense:** 5′-GACTCATCGTACTCCTGCTGCTG-3′	FQ228940.1		60
**NR**_**2**_ **A**	**Sense:** 5′-CCGATAATCCTTTCCTCCACA-3′ **Anti-sense:** 5′-TTGTAAGGGTCCGAGGGACAT-3′	XM_017596985.1		57–60
**NR**_**2**_ **B**	**Sense:** 5′-ATGTCTCAGACATCTCCACGCACA-3′ **Anti-sense:** 5′-TGCTGTTTCCTCCTCTTGGC-3′	XM_017592439.1		60–63

### Evaluation of genotoxicity in the isolated brain regions

#### Preparation of single cell suspension from brain regions

Small pieces of brain regions (PFC, hippocampus, and striatum) from each group were quickly minced into approximately 1–3 mm pieces while immersed in 1 ml ice-cold phosphate-buffered saline (PBS). The single cell suspension from each brain region was made by pipetting. The cell suspensions were centrifuged at 60-70 rpm for 5 min and stored at −80 °C. The pellets were resuspended in 1% low-melting point agarose at a ratio of (1:9). The tissue suspension was pipetted and casted onto the comet slide. The slides were immersed into freshly prepared cold lysing solution (2.5 M NaCl, 100 mM EDTA, 10 mM Trizma base), 1% Triton X-100, 10% di-methyl sulphoxide (DMSO), and protinase K (10 µg/ml) and incubated overnight at 37 °C.

#### Comet assay (Single cell gel electrophoresis assay)

Alkaline comet assay was performed according to the protocol of Swain & Rao (2011). For each slide, 100 randomly comets were analyzed using a 3-CCD camera attached a Leitz Orthoplan epifluorescence microscope equipped with an excitation filter of 515–560 nm and a barrier filter of 590 nm. Images were scored for the following comet parameters; the comet tail length, the tailing ratio, and tail moment by using the comet assay IV software.

### Histopathological investigations of the isolated brain regions

The fixed brain tissues were dehydrated using ascending concentrations of ethyl alcohol, cleared, and then embedded in paraffin wax for 2 h to form blocks. Paraffin-embedded brain tissues were sectioned, de-waxed, hydrated and stained with hematoxylin and eosin for histological examination (Drury & Wallington, 1980; Essawy et al., 2019).

### Immunohistochemical investigations of estrogen receptors immunoreactive cells in the isolated brain regions

Paraffin-embedded brain sections were mounted on charge slides and incubated at 37 °C overnight on the benchtop. Deparaffinized sections were incubated with Ultra V Block reagent for 30 min, and then incubated overnight at 4 °C with estrogenic receptor primary antibody. Finally, the sections were incubated with UltraVision ONE HRP Polymer for 30 min at room temperature. The peroxidase reaction was visualized with 3,3′-diaminobenzidine (DAB) chromogen mixture. Sections were counterstained with hematoxylin. A five fields from each section were selected and analyzed for the positive ER-immunostaining intensity using the Image J analyzer (version 5.1) (Petrosyan, Tamayo & Joseph, 2002; Shi et al., 1999).

### Statistical analysis

Statistical analysis was carried out using Statistical Package for the Social Sciences software (SPSS, version 16.0). The results were presented as the mean ± S.E of the mean. Statistically significant differences in parametric data were determined by one-way ANOVA followed by Tukey’s HSD and Games-Howell multiple comparison test. However, statistical significance levels in non-parametric data were determined by the Kruskal-Wallis test followed by the Mann–Whitney U test. Values were considered significantly different when *p* ≤ 0.05.

## Results

### Biochemical findings

Data represented in Table 2 showed the concentration of NO and the activities of glutaminase (GA) and glutamine synthase (GS) in different regions of the brain of the treated rats. A significant (*P* ≤ 0.05) increase in NO concentration and significant decrease in the activities of GA and GS was observed in the PFC, hippocampus, and striatum of BPA-treated group compared with control. Co-administration of ASIV or *A. spinosus* saponin with BPA significantly (*P* ≤ 0.05) reduced the concentration of NO in the mentioned brain regions of these two groups compared with BPA-treated group. The co-administration of ASIV with BPA showed significant elevation in the activity of GA in both hippocampus and striatum regions and significant elevation in the activity of GS only in the hippocampus region compared with BPA-group. While non-significant reduction in the activities of both GA and GS were observed in the other brain regions of BPA+ASIV-treated group. Also, BPA+*A. spinosus* saponin-group showed significant (*P* ≤ 0.05) increase in the activities of GA of striatum and GS of hippocampus and non-significant increase in both enzymes of other brain regions.

**Table 2:** Effect of BPA alone or in combination with ASIV or *A. spinosus* saponin on the concentration of nitric oxide and the enzymatic activities of glutaminase and glutamine synthetase in the isolated brain regions.

***Parameters***	***Experimental Groups***			
	**Control**	**BPA**	**BPA+ASIV**	**BPA+** ***A. spinosus*** **Saponin**
**NO** ***(*μ*mol/mg protein)***	
PFC	0.24 ± 0.01	0.31 ± 0.01^a^	0.25 ± 0.01^b^	0.26 ± 0.01^b^
Hippocampus	0.29 ± 0.01	0.63 ± 0.06^a^	0.34 ± 0.04^b^	0.27 ± 0.03^b^
Striatum	0.24 ± 0.01	0.42 ± 0.03^a^	0.27 ± 0.01^b^	0.28 ± 0.02^b^
**Glutaminase activity** ***(mM/min/mg protein)***	
PFC	389 ± 35.85	285 ± 15.30^a^	294 ± 24.42^a^	335 ± 9.02
Hippocampus	259 ± 7.46	235 ± 7.36^a^	313 ± 9.83^a^^,^^b^	254 ± 5.82
Striatum	1227 ± 11.85	405 ± 24.24^a^	1557 ± 124^a^^,^^b^	1064 ± 8.92^b^
**Glutamine synthase activity** ***(mM/min/mg protein)***	
PFC	22.08 ± 0.89	18.99 ± 1.16^a^	21.28 ± 0.74	18.59 ± 0.64^a^
Hippocampus	23.78 ± 0.98	17.71 ± 1.59^a^	24.07 ± 0.74^b^	23.53 ± 2.65^b^
Striatum	37.65 ± 1.27	28.77 ± 1.45^a^	33.09 ± 1.35	31.59 ± 2.81^a^

**Notes.**

Values are expressed as means of 7 rats ±  SE. Statistical significant test for comparison was done by ANOVA followed by post hoc Tukeys HSD multiple comparison test.

a The mean values are significantly different in comparison with the control group at *p* ≤ 0.05.

b The mean values are significantly different in comparison with the BPA-group at *p* ≤ 0.05.

The One-way ANOVA followed by Tukey’s HSD test revealed a significant (*P* ≤ 0.05) decrease in the concentration of glutamine (Gln) of the three brain regions of BPA-group compared with control. On the contrary, the glutamate concentration of both PFC and hippocampus was significantly increased in the BPA-group. Co-administration of ASIV with BPA leads to significant (*P* ≤ 0.05) increase in the Gln concentration of striatum region compared with BPA-group, while Gln or Glu concentrations showed non-significant changes in the other brain regions of this group. The co-treatment of BPA-group with *A. spinosus* saponin significantly (*P* ≤ 0.05) reduced the Glu concentration in the PFC and hippocampus regions and significantly elevated the Gln concentration in the PFC and striatum regions of this group compared with BPA-group (Table 3).

**Table 3:** Effect of BPA alone or in combination with ASIV or *A. spinosus* saponin on the concentrations of glutamate and glutamine in the isolated brain regions.

***Parameters***	***Experimental Groups***			
	**Control**	**BPA**	**BPA+ASIV**	**BPA+** ***A. spinosus*** **Saponin**
**Glutamate** ***(*μ* mol/g tissue)***	
PFC	4.98 ± 0.40	6.47 ± 0.39^a^	5.57 ± 0.36	3.98 ± 0.12^b^
Hippocampus	10.34 ± 0.82	14.56 ± 0.73^a^	12.48 ± 1.08	10.12 ± 0.39^b^
Striatum	14 ± 1.23	11.76 ± 0.76	13.13 ± 0.79	12.52 ± 0.53
**Glutamine** ***(*** ****μ*mol/g tissue*** ***)***	
PFC	4.46 ± 0.40	2.67 ± 0.09^a^	2.75 ± 0.14^a^	3.75 ± 0.29^b^
Hippocampus	10.60 ± 0.71	4.45 ± 0.35^a^	5.48 ± 0.41^a^	5.69 ± 0.44^a^
Striatum	4.42 ± 0.40	2.32 ± 0.10^a^	4.80 ± 0.42^b^	4.43 ± 0.40^b^

**Notes.**

Values are expressed as means of 7 rats ±  SE. Statistical significant test for comparison was done by ANOVA followed by post hoc Tukey’s HSD multiple comparison test.

a The mean values are significantly different in comparison with the control group a *p* ≤ 0.05.

b The mean values are significantly different in comparison with the BPA-group at *p* ≤ 0.05.

**Figure 1:**
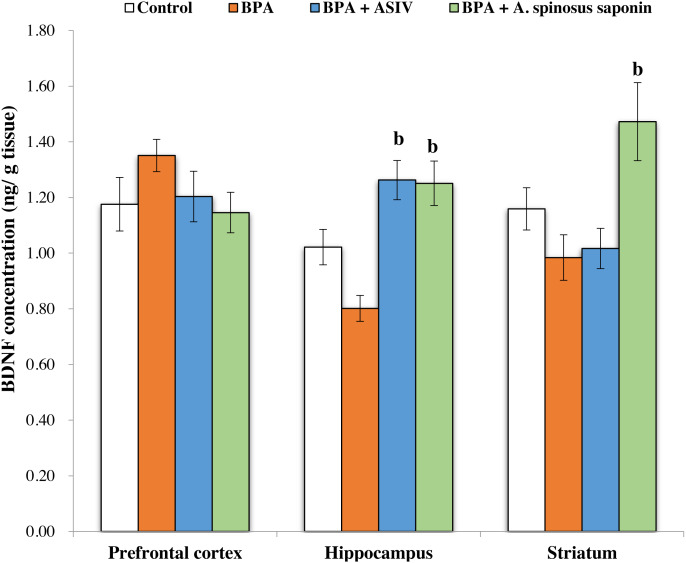
Effect of treatments on brain derived neurotrophic factor (BDNF) concentration in different regions of brain in the studied groups. All the data were analyzed using one-way ANOVA followed by Tukey’s HSD test. Values are expressed as means of six rats ±SE as compared to control group (A), BPA-treated group (B) at *P* < 0.05. BPA; bisphenol A, ASIV; astragaloside IV and *A. spinosus* saponin; *Astragalus spinosus* saponin.

The data represented in Fig. 1 showed non-significant reduction in the concentration of derived neurotrophic factor (BDNF) in the brain PFC, hippocampus and striatum regions of BPA-group compared with control. The ASIV or *A. spinosus* saponin significantly (*P* ≤ 0.05) increased the BDNF in the hippocampus regions while only *A. spinosus* saponin can significantly increase BDNF in the striatum region of the brain compared with BPA-group. Non-significant changes in the levels of BDNF were observed between the PFC of the four experimental groups.

### Molecular findings

**Figure 2:**
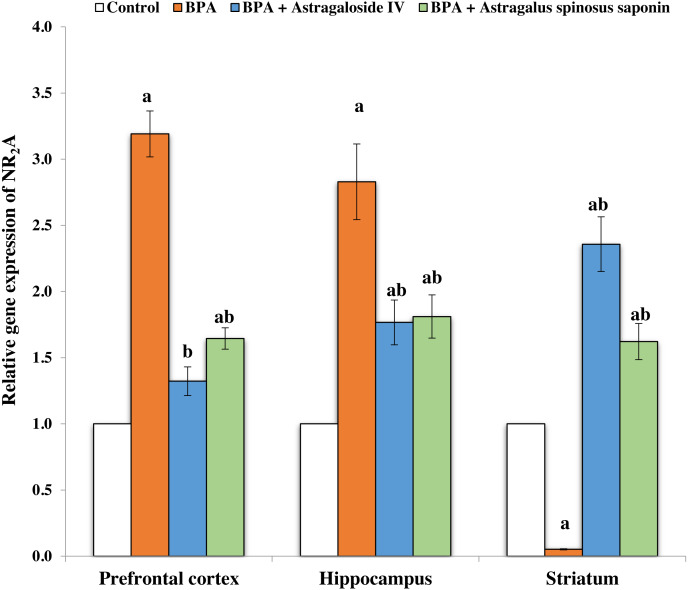
Effect of treatments on the relative mRNA expression levels of NR_2_A in different regions of brain in the studied groups. β-actin serves as internal control. All the data were analyzed using one-way ANOVA followed by Tukey’s HSD test. Values are expressed as means of six rats ± SE as compared to control group (A), BPA-treated group (B) at *P* < 0.05. BPA; bisphenol A, ASIV; astragaloside IV and *A. spinosus* saponin; *Astragalus spinosus* saponin.

**Figure 3:**
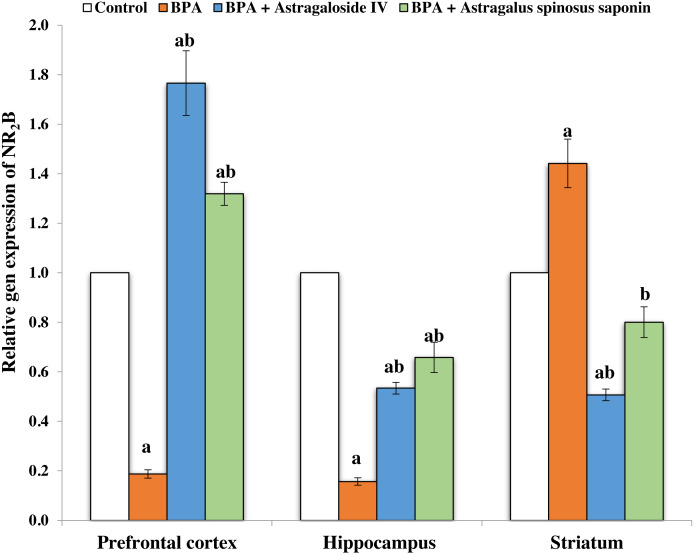
Effect of treatments on the relative mRNA expression levels of NR_2_B in different regions of brain in the studied groups. β-actin serves as internal control. All the data were analyzed using one-way ANOVA followed by Tukey’s HSD test. Values are expressed as means of six rats ± SE as compared to control group (A), BPA-treated group (B) at *P* < 0.05. BPA; bisphenol A, ASIV; astragaloside IV and *A. spinosus* saponin; *Astragalus spinosus* saponin.

Figures 2 and 3 demonstrates the mRNA expression level of NR_2_A and NR_2_B receptors respectively in the experimentally studied groups. In the PFC and hippocampus regions of BPA-treated group, the NR_2_A mRNA expression levels were significantly (*P* ≤ 0.05) upregulated and the NR2B expressions were significantly downregulated compared with control. The opposite finding was observed in the striatum region of the same group; the NR_2_A significantly (*P* ≤ 0.05) downregulated and the NR_2_B significantly upregulated. The co-treatment of BPA-group with ASIV or *A. spinosus* saponin significantly revert the effect of BPA on the expression levels of both NR_2_A and NR_2_B compared with BPA-treated group.

### Genotoxicity results

**Figure 4:**
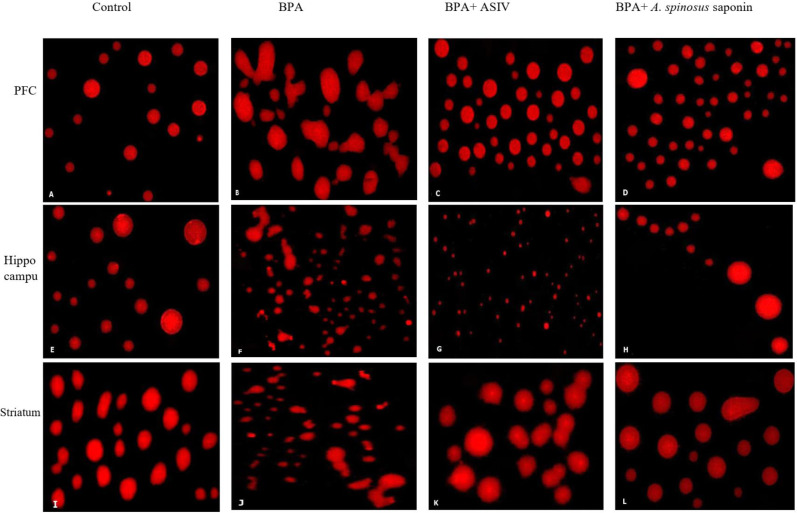
Photomicrographs of DNA damage in the PFC, hippocampal and striatal sections of brain in the studied groups. (A, E & I) Sections from control rats. (B, F & J) Sections from BPA-treated rats, showing increase DNA damage and comet tail migration. (C, G & K) Sections from rats treated with BPA+ ASIV. (D, H & L) Sections from rats treated with BPA+ *A. spinosus* saponin showed less damage of DNA. All images are at 400X magnifications.

The results of comet assay, which are expressed as tail length, tail moment and percentage of tailing ratio for the studied brain regions were represented in Table 4 and Fig. 4. The cells of PFC, hippocampus and striatum regions of rats exposed to BPA showed significant increase in the DNA damage as indicated by the significant (*P* ≤ 0.05) elevation in the mean values of tail length, tail moment and percentage of tailing ratio compared with that of control. However, co-treatment of BPA-group with ASIV or *A. spinosus* saponin significantly reduced the mean values of comet parameters induced by BPA in these specified regions compared with BPA-group.

**Table 4:** Effect of BPA alone or in combination with ASIV or *A. spinosus* saponin on DNA damage in the isolated brain regions using comet assay.

***Parameters***	***Experimental Groups***			
	**Control**	**BPA**	**BPA+ASIV**	**BPA+** ***A. spinosus*** **Saponin**
**Tail length** ***(*μ*m)***	
PFC	19.57 ± 0.39	70.82 ± 2.06^a^	16.41 ± 0.20^a^^,^^b^	17.58 ± 0.37^a^^,^^b^
Hippocampus	26.97 ± 0.65	41.41 ± 1.27^a^	10.49 ± 0.31^a^^,^^b^	21.08 ± 0.71^a^^,^^b^
Striatum	14.21 ± 0.29	56.14 ± 1.97^a^	21.31 ± 0.26^a^^,^^b^	30.21 ± 0.47^a^^,^^b^
**Tail moment** ***(arbitrary unit)***	
PFC	0.99 ± 0.06	6.37 ± 0.38^a^	1.83 ± 0.06^a^^,^^b^	1.70 ± 0.13^a^^,^^b^
Hippocampus	1.98 ± 0.12	12.42 ± 1.06^a^	0.93 ± 0.07^a^^,^^b^	2.54 ± 0.20b
Striatum	3.22 ± 0.23	6.28 ± 0.35^a^	1.51 ± 0.13^a^^,^^b^	2.15 ± 0.13^a^^,^^b^
**Tailing ratio** ***(%)***	
PFC	51.91 ± 2.86	130 ± 3.54^a^	73.74 ± 2.82^a^^,^^b^	52.30 ± 3.72^b^
Hippocampus	47.22 ± 3.55	107 ± 5.57^a^	57.82 ± 4.42^b^	44.75 ± 3.51^b^
Striatum	44.65 ± 3.94	115 ± 3.74^a^	64.50 ± 3.56^a^^,^^b^	64.39 ± 3.03^a^^,^^b^

**Notes.**

Values are expressed as means ±  SE; *n* = 6 with 100 observations for each group. Statistical significant test for comparison was done by ANOVA followed by post hoc Tukey’s HSD multiple comparison test.

a The mean values are significantly different in comparison with the control group at *p* ≤ 0.05.

b The mean values are significantly different in comparison with the BPA-group at *p* ≤ 0.05.

### Histopathological findings

**Figure 5:**
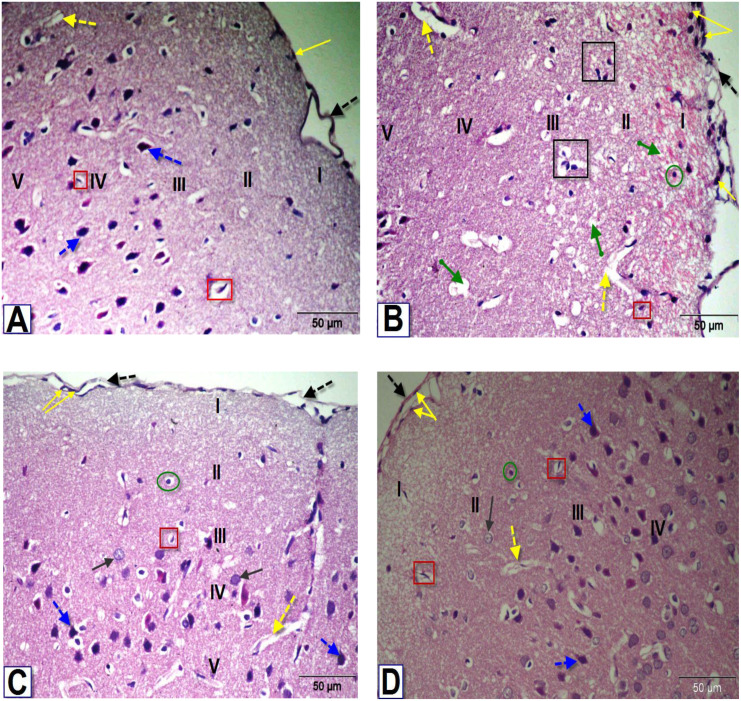
Photomicrographs of coronal sections in the PFC of male rats. (A) Section from control rat, showing normal meninges; dura mater with low cellularity (black dotted arrow), thin pia mater (yellow arrow), outer molecular layer (I), external granular layer (II), external pyramidal layer (III), inner granular layer (IV) and inner pyramidal layer (V). Blood capillaries (yellow dotted arrow), astrocytes (red square) and pyramidal cells (blue dotted arrows). (B) Section from BPA-treated rat, showing dura mater with hypercellularity, thickened pia mater, thick disorientation of the cellular layers with cellular infiltration, congested outer molecular layer with astrocytes (green circle), apoptotic and degenerated cells (black square), dilated blood vessels (yellow dotted arrows) and several vacuoles (green arrow). (C & D) Sections from rats treated with BPA+ ASIV or *A. spinosus* saponin respectively, showing dura mater, thin pia mater, moderate normal granular cells (black arrow), pyramidal cells, blood capillaries and astrocytes (H&E, X400).

**Figure 6:**
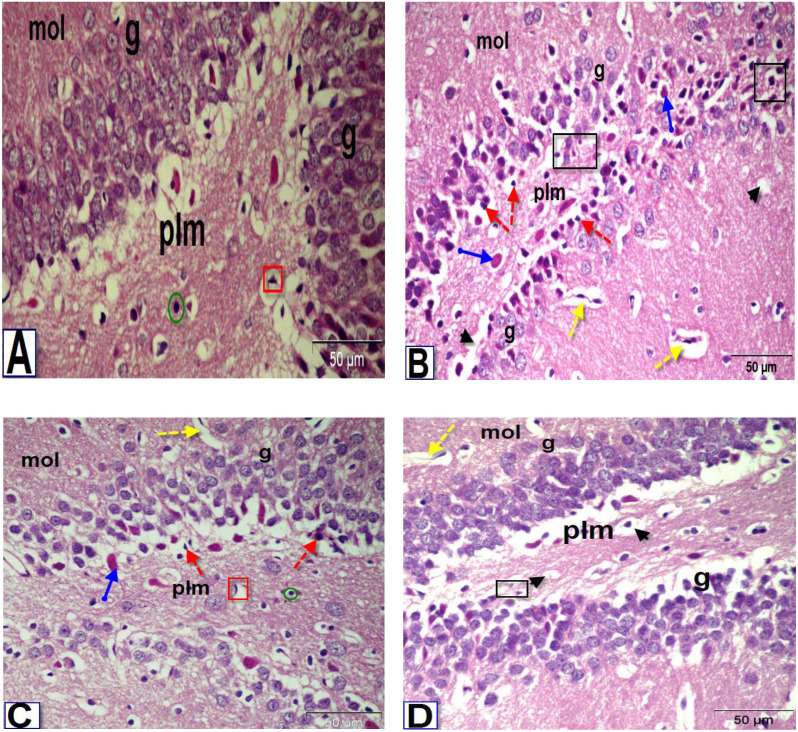
Photomicrographs of coronal sections in the dentate gyrus of male rats. (A) Section from control rat, showing granular (g), polymorphic (plm) and molecular (mol) layers. The granular layer contains intense columns of mature neurons (g), while the polymorphic layer showed neuroglial cells (green circle) and large number of astrocytes (red square). (B) Section from BPA-treated rat, showing less intensity granular layer and degenerated neurons (black square) with darkly stained pyknotic nuclei (red dotted arrows) surrounded by a large perinuclear space (arrowhead), presence of red neurons in granular and polymorphic layers (blue arrow) and abnormal dilated blood vessels (yellow dotted arrows) in the molecular layer. (C & D) Sections from rats treated with BPA+ ASIV or *A. spinosus* saponin respectively, showing increase in the density of granular cells, presence of few cells with darkly stained nuclei and vacuolated cytoplasm (arrowhead), blood vessels (yellow dotted arrows), red neurons and astrocytes (H&E, X400).

**Figure 7:**
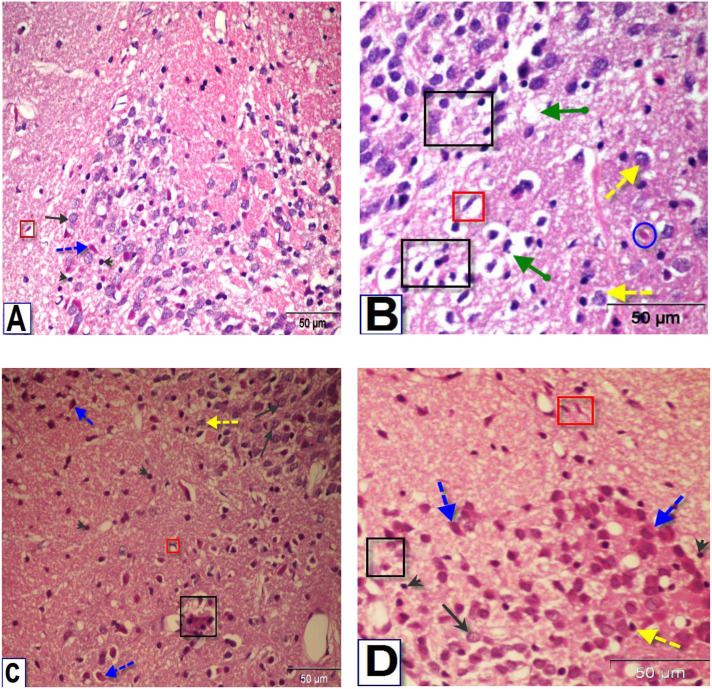
Photomicrographs of coronal sections in the striatum of male rats. (A) Section from control rat, showing normal structure of neurons (black arrows) and glial cells (arrowhead). (B) Section from BPA-treated rat, showing neuronal degeneration (yellow dotted arrow), disorganization and encephalomalacia (black square), neuronal cells with pyknotic nuclei, pericellular edema and vacuoles (green arrow), red neuron (blue circle) and astrocytes (red square). (C & D) Sections from rats treated with BPA+ ASIV or *A. spinosus* saponin respectively, showing normal neurons, few neurons with pyknotic nuclei and astrocytes (H&E, X400).

Microscopic examination of brain sections from control rats (Figs. 5, 6 and 7A) showed normal histological structure of the PFC, dentate gyrus (DG) and striatum. However, the histopathological examination of the PFC of BPA-treated rat (Fig. 5B) showed dura mater with hyper-cellularity, thickened pia mater, disorientation of the layers and cellular infiltration. Moreover, pathological changes were observed in layers of the cortex, including congested outer molecular with large number of glial cells, presence of many apoptotic nuclei and degenerated neurons, presence of dilated blood vessels and vacuolated cytoplasm. On the contrary, the PFC sections of rats treated with BPA+ ASIV or *A. spinosus* saponin (Figs. 5C and 5D, respectively) exhibited improvement in dura mater, thin pia mater, restoration in the layers of the cortex and normal blood capillaries.

The most obvious changes in the DG region of BPA-treated rat (Fig. 6B) were the presence of degenerated shrunken granule cells, pyknotic nuclei, dilated blood vessels and vacuolated cytoplasm. The histological examination DG of both BPA+ ASIV and BPA+ *A. spinosus* saponin groups (Figs. 6C and 6D, respectively) showed preservation of neurons and normal blood vessels. Few numbers of pyramidal cells with darkly stained pyknotic nuclei and few vacuoles.

The examination of the striatal region of BPA-treated group (Fig. 7B) revealed neuronal degeneration associated with pericellular edema, encephalomalacia and vacuolization. Also, neuronal cells with distorted and darkly stained pyknotic nuclei were observed. Sections of the striatal region of rats treated with BPA+ ASIV and BPA+ *A. spinosus* saponin (Figs. 7C and 7D, respectively) showed normal structure of different types of neurons, few degenerated neurons with pyknotic nuclei, vacuolated cytoplasm, and few glial cells.

### Immunohistochemical results

Our results showed a significant (*P* ≤ 0.05) increase in the intensity of ER immunoreactive cells in the PFC after BPA exposure, whereas a marked decrease in the hippocampus and striatum was recorded compared with control. The number of ER-positive immunostaining per field was significantly (*P* ≤ 0.05) decreased in the three brain regions of BPA-treated group compared with that of control. On the other hand, the co-treatment with ASIV or *A. spinosus* saponin significantly revert the effect of BPA and markedly improve the intensity of ER immune-reactivity compared with BPA-treated group (Table 5).

## Discussion

Data in the present study illustrated that NO concentration was markedly increased in the PFC, hippocampus, and striatum in BPA-treated animals. These finding are in agreement with the results of Akintunde, Farouk & Mogbojuri (2019) who reported that BPA causes marked elevation in NO level and altered neuronal functions. The enhanced NO production may be due to activation of NMDA receptors. This activation may stimulate the increase in the intracellular Ca^2+^ which binds to calmodulin and induces various enzymes, including nitric oxide synthase (NOS) (Renuka, Vijayakumar & Ramakrishnan, 2016). Co-administration of ASIV or *A. spinosus* saponin considerably reverted the NO concentration in the PFC, hippocampal and striatal regions. These results are in line with those of Wang et al. (2019) who revealed that ASIV alleviates the oxidative stress in mammary epithelial cells. Other authors showed that ginsenosides Rb1 and Rb3 could inhibit the overproduction of NO, which routinely follows Glu neurotoxicity, and diminished the influx of calcium (Sun et al., 2015).

Glutaminase (GA) and glutamine synthase (GS) are multifunctional enzymes of the Glu-Gln cycle and are crucial for ammonia trafficking and regeneration of Glu (Bae et al., 2013). Our results showed that administration of BPA significantly decreased the specific activities of GA and GS in the PFC, hippocampus, and striatum regions. The underlying mechanism behind GS suppression is that Gln accumulation could inhibit the GS activity. In addition, the reduction in GS activity could stem from a direct effect of BPA or indirectly through the reduction of ATP availability since GS is an energy demanding enzyme (Abulseoud et al., 2017). Suppression of GA activity in the present study may be due to the presynaptic mechanism of impaired long-term potentiation (LTP) by BPA intoxication (Hu et al., 2017). Thus, the decreased activity of GA is a prospective inhibition to attenuate excitatory activity of BPA (Mingote et al., 2016).

A marked increase in Glu concentration was observed in both PFC and hippocampus regions accompanied with insignificant change in the striatum of BPA-treated rats. Bisphenol A may impair components of the Glu-Gln cycle leading to increase in Glu uptake, which induces the accumulation of extracellular Glu level and reduces the capacity of astrocytic Glu transporter (Szpetnar et al., 2016). Changes in Glu signaling in the striatum may be due to the low level of Glu transporters in the presynaptic terminals (Barakauskas et al., 2010). The decrease of Gln concentration in the present study was noticed in all brain regions of BPA-treated rats. These results agree with those of Khadrawy et al. (2016). Reduced Gln concentration could reflect the inhibition of GS activity which causes reduction in neuronal energy level and suppresses the synthesis of Gln (Szpetnar et al., 2016). Finally, the disturbances of Glu-Gln cycle could induce the elevation of Glu and the decrement of Gln concentration (Robinson et al., 2015). On the other hand, co-administration of ASIV or *A. spinosus* saponin with BPA markedly attenuate the effect of BPA on GA, GS, Glu and Gln in the PFC, hippocampus, and striatum. These results are in consistent with that of Naseri et al. (2019). One explanation for the inhibitory effects of ASIV on overall membrane excitability is the blockade of voltage-gated K^+^ and Na^+^ currents, producing a subsequent reduction of Ca^2+^-dependent excitatory transmitter release (Sun et al., 2015). The potential protective mechanisms of saponin include its antioxidant, anti-inflammatory, anti-apoptotic, downregulation of excitotoxicity, induction of excessive Ca^2+^ influx into nerve cells and restoration of cellular ATP levels (Xie et al., 2018).

The current study showed that BPA markedly decreased BDNF concentration in the hippocampal and striatal regions and insignificantly affect it in the PFC. The decreased level of BDNF in the hippocampus may be depend upon the NMDA receptors, which can be a target for the modulation of BDNF expression and its action on synaptic plasticity (Roceri et al., 2002). Dissimilar effects of BPA on BDNF levels in different brain regions may be related to different stages of development in each brain area and diverse metabolic pathways which may modulate the mode of action of BPA (Wang et al., 2016). Co-administration of ASIV or *A. spinosus* saponin ameliorate the effect of BPA and restored the level of the BDNF to the control levels. The underlying mechanisms of saponin could encompass the modulation of several synaptic regulators, including BDNF/TrkB/NF-κB pathway, NMDA, Glu and estrogen (Duman et al., 2016; Zhang et al., 2019).

BPA exposure in the current study resulted in upregulation of NR_2_A mRNA expression levels in the PFC and hippocampus and its downregulation in the striatum. In contrary, the NR_2_B mRNA expression level was downregulated in PFC and hippocampus regions and upregulated in striatum brain region of BPA-treated rats. These results are in accordance with those of Khadrawy et al. (2016) who suggested that the upregulation of NR_2_A mRNA expression level may be related to elevation in the excitatory amino acids such as Glu following the exposure to BPA. This resulted in overstimulating glutamatergic NMDARs whose prolonged depolarization induces a massive influx of Ca^+2^ (Khadrawy et al., 2016). The inhibition in the expression of NMDARs and the alteration of their subunit’s composition may be a strategy by which the brain attempts to minimize the state of excitotoxicity induced by BPA (Khadrawy et al., 2016). The co-treatment of rats with ASIV or *A. spinosus* saponin revert the effect of BPA on the expression levels of NR_2_A and NR_2_B mRNA in different brain regions. This could be due to direct block of the NMDA glutamate receptors by ASIV, reduction of Glu release, spontaneous neuronal excitabilities and the neuronal firing *via* blocking of voltage-gated K^+^ and Na^+^ channels (Li et al., 2017; Sun et al., 2015). Moreover, saponins significantly protected NMDA-induced neuronal death *via* a competitive interaction with the glycine binding site of NMDARs (Sun et al., 2015). It was also shown that saponin displays a potent inhibitory activity against the excitotoxic damage caused by NMDARs through blocking the combination of the NMDAR with Glu to prevent Glu toxicity (Zhang et al., 2014).

The genotoxic analysis by comet assay confirmed that BPA induced a marked DNA damage in the studied brain regions as indicated by the marked increase in the tail length, tail moment and tailing ration. These results are in agreement with the findings of Zhang et al. (2020). The incidence to oxidative stress, initiation of ROS generation and the depletion of antioxidant enzymes could be the possible causes of BPA genotoxicity (George & Rupasinghe, 2018). Besides, BPA is an EDC which exerts an estrogenic effect that may interfere with normal estrogenic signaling (Fawzy et al., 2018). Administration of ASIV or *A. spinosus* saponin was able to restore the comet scores near to control values. These findings are concomitant with the results of Khalil, Roshdy & Kassem (2016) who illustrated that this attenuation may be related to the efficiency of saponin as a free radical-scavenging and decreased rate of DNA damage.

**Table 5:** Effect of BPA alone or in combination with ASIV or ***A. spinosus*** saponin on the immunohistochemical examination of estrogen receptor in the isolated brain regions.

***Parameters***	***Experimental Groups***			
	**Control**	**BPA**	**BPA+ASIV**	**BPA+** ***A. spinosus*** **Saponin**
**Intensity of ER-immunostaining**	
PFC	181 ± 1.57	209 ± 1.12^a^	183 ± 1.57^b^	201 ± 1.98^a^^,^^b^
Hippocampus	197 ± 2.17	174 ± 2.41^a^	190 ± 2.08^b^	194 ± 2.38^b^
Striatum	163 ± 1.14	153 ± 4.86	158 ± 1.02^a^	163 ± 2.71
**Number of ER-positive immunostaining per field**	
PFC	23.67 ± 0.92	13.20 ± 0.79^a^	22.67 ± 1.52^b^	25.40 ± 2.04^b^
Hippocampus	26.67 ± 0.67	17 ± 0.68^a^	26.67 ± 0.84^b^	31.80 ± 0.79^a^^,^^b^
Striatum	34 ± 1.83	21.40 ± 0.71^a^	31.50 ± 0.62^b^	31.33 ± 1.75^b^

**Notes.**

Values are expressed as means ± SE; *n* = 5 fields with 7 observations/field for each group. Statistical significant test for comparison was done by ANOVA followed by post hoc Tukey’s HSD multiple comparison test.

a The mean values are significantly different in comparison with the control group at *p* ≤ 0.05

b The mean values are significantly different in comparison with the BPA-group at *p* ≤ 0.05

The present investigation identified many histopathological alterations in the PFC, DG, and striatum of rats exposed to BPA, and suggest its possible cytotoxic activity. Other researchers demonstrated similar cytotoxic effects for BPA in the neurons of hippocampus (Abd Elaziz & Laag, 2018). Due to the lipophilic nature of BPA, it could easily penetrate the BBB and accumulate inside the brain. Accumulation of BPA in brain results in a condition of oxidative stress and DNA damage which can provoke the degeneration of nerve cells (Abd Elkader et al., 2021). The appearance of vacuolated pericellular spaces was mostly due to shrinkage of neurons and withdrawal of their cytoplasmic process’s secondary to disintegration of the cytoskeletal elements of these cells (Abd Elaziz & Laag, 2018). However, treatment with ASIV or *A. spinosus* saponin efficiently improved the histological alterations in different brain regions. In agreement with these finding, Huang et al. (2017) verified that administration of ASIV or saponin effectively suppressed the oxidative stress and inflammation in the brain, thereby inhibiting the destructive changes and aberrant apoptosis of developing nerve cells.

Interestingly, our results showed that exposure to BPA reduces the expression level of ER-α. Xu et al. (2014) reported that exposure to BPA during the early postnatal period altered the ER-α expression in the developing hippocampus of male rats *via* ER-dependent pathways. They suggested that BPA modulates the synthesis and phosphorylation of ER-α and disrupting the nuclear translocation of ER-α during this period of development. Co-treatment with BPA+ASIV or *A. spinosus* saponin significantly ameliorated the ER-α expression level toward control values. Structurally, saponins like steroids can selectively bind to ERs and modulate relevant gene expression to produce estrogenic and/or anti-estrogenic actions (Wang et al., 2018). Moreover, they could exert their mode of action by activating transcription and nuclear translocation of ERs (Shen et al., 2014).

## Conclusion

In the light of present results, it could be concluded that BPA administration induced a broad spectrum of biochemical, genetic, histopathological and immunohistochemical alterations, as well as DNA damage in the PFC, hippocampal, and striatal brain regions. Supplementation with ASIV or *A. spinosus* saponin exhibit a novel neuroprotective approach against BPA-induced neurotoxicity in the developing male rats. The ameliorative effects of ASIV or *A. spinosus* saponin could be attributed to their antioxidant, anti-inflammatory, and anti-apoptotic activity as well as to activating signaling pathways related to synaptic plasticity.

##  Supplemental Information

10.7717/peerj.11930/supp-1 Supplemental Information 1 Raw data All measurements performed in the brain regions of individual rats in all groups during the work.

10.7717/peerj.11930/supp-2 Supplemental Information 2 Author Checklist

## Additional Information and Declarations

### Data Availability

The following information was supplied regarding data availability: The raw measurements are available in the Supplementary File.
